# Supplementary material for: Mercury evidence from southern Pangea terrestrial sections for end-Permian global volcanic effects
Source: Nat Commun. 2023 Jan 3;14:6. doi: 10.1038/s41467-022-35272-8 (PMC9810726; doi:10.1038/s41467-022-35272-8)
Supplement: Supplementary file 1 — Supplementary Information [file 41467_2022_35272_MOESM1_ESM.pdf]

# Description of Supplementary Files

File Name: Supplementary Information for “Mercury evidence from southern Pangea terrestrial sections for end-Permian global volcanic effects”

Jun Shen, Jiubin Chen, Jianxin Yu, Thomas J. Algeo, Roger M.H. Smith, Jennifer Botha, Tracy D. Frank, Christopher R. Fielding, Peter D. Ward, Tamsin A. Mather

Description: Supplementary Notes (1-2), Supplementary Figures (1-6), Supplementary Table (1), and Supplementary References.

# Supplementary Notes

## Supplementary Note 1 | Host phases of Hg in the sediments

Robust use of Hg as a volcanic proxy requires an understanding of its sedimentary host phases in the target formation. Most studies have assumed organic matter to be the dominant host of Hg, relying on mercury to total organic carbon ratios (Hg/TOC) to assess Hg enrichments in ancient successions[1,2]. However, Hg also can be associated with the sulfide[2-4] or clay fractions of the sediment[2, 5], invalidating TOC normalization of Hg concentration data. The environmental controls influencing Hg uptake by various sedimentary phases are not well understood. Hg-S complexes have high stability constants and can dominate Hg speciation under reducing conditions where $H_2S$ is present in porewaters[2-4]. Clay minerals are also capable of adsorbing Hg, leading to significant Hg enrichments in some shales[2, 5].

For the Sydney Basin (Supplementary Fig. 2), Hg concentrations vary strongly throughout the two study cores, ranging from <10 ppb to >100 ppb (Supplementary Fig. 2b, k). TOC also exhibits large variations, with lower values (e.g., <0.5%) in the Lower Triassic and higher values (e.g., >10%) near the Permian–Triassic transition and in the Upper Permian (Supplementary Fig. 2c, l). These cores have low total sulfur (TS) contents (<0.3 %), with a few minor peaks (e.g., >0.5%) in the Capitanian (Supplementary Fig. 2d, m). Aluminium (Al) values are relatively stable, ranging from 6 % to 12% for most samples (Supplementary Fig. 2e, n). Hg exhibits a stronger correlation to TOC ($r$ = +0.60, $n$ = 70, $p$ < 0.01, and $r$ = +0.79, $n$ = 59, $p$ < 0.01, for Bunnerong and Eveleigh, respectively; Supplementary Fig. 4a) than to TS ($r$ = +0.29, $n$ = 70, $p$ > 0.05, and $r$ = +0.44, $n$ = 56, $p$ < 0.01; Supplementary Fig. 4b) or Al ($r$ = +0.05, $n$ = 40, $p$ > 0.05, and $r$ = +0.08, $n$ = 54, $p$ > 0.05; Supplementary Fig. 4c), indicating that organic matter is the dominant host of Hg. For this reason, Hg/TOC is a suitable normalization for Hg at Bunnerong and Eveleigh. A minimum TOC content of 0.2 % was used for TOC normalizations in order to avoid instability associated with a small denominator[6]. Samples yielding high Hg/TOC (e.g., >30 ppb/%) have TOC values that range from <0.2 % to >5 %, suggesting that TOC variation did not generate spurious spikes in the Hg/TOC profile.

For the Karoo Basin (Supplementary Fig. 3), Hg concentrations are lower in the background beds than in the extinction interval, where Hg rises to >10 ppb and >20 ppb in the Ripplemead and Bethulie sections, respectively (Supplementary Fig. 3b, j). Both TOC (<0.3 %, Supplementary Fig. 3c, k) and TS (0.06 %, Supplementary Fig. 3d, l) are low for most samples in these sections. Al values are relatively stable, ranging from 7 % to 9 % for most samples (Supplementary Fig. 3e, m). Hg exhibits no or weak correlations to TOC ($r = + 0.02$, $n = 127$, $p > 0.05$, and $r = –0.03$, $n = 152$, $p > 0.05$, for Ripplemead and Bethulie, respectively; Supplementary Fig. 4d), TS ($r = –0.12$, $n = 127$, $p > 0.05$, and $r = –0.07$, $n = 152$, $p > 0.05$; Supplementary Fig. 4e), and Al ($r = –0.16$, $n = 60$, $p > 0.05$, and $r = + 0.00$, $n = 99$, $p > 0.05$; Supplementary Fig. 4f), rendering uncertain which sedimentary phase is the dominant host of Hg in these sections. Despite this uncertainty, we chose to utilize Hg/TOC normalizations rather than Hg/TS or Hg/Al normalizations, which has the advantage of maintaining equivalency of data display with the Sydney Basin cores, in which the organic fraction is unambiguously the main host of Hg, as well as with the results of many other studies (see review by Grasby et al.[1]).

## Supplementary Note 2 | Age-thickness model of Bunnerong core

An age-thickness model was developed for the Bunnerong core based on a combination of radiometric ages for volcanic ash beds and palynological data[7-10]. This model yielded sediment accumulation rates of ~63 m/Myr for the marine lower succession (e.g., shelf/delta/coastal-plain) and ~108 m/Myr for the terrestrial upper succession (e.g., coastal- and alluvial-plain)[9] (Supplementary Fig. 6). The timespan between the onset of Hg enrichment (at ~820 m) and the onset of the negative carbon isotope excursion linked to the Permian–Triassic transition (at ~800 m) is estimated to have been between 180 and 320 kyr (Supplementary Fig. 6).

## Supplementary Figures:

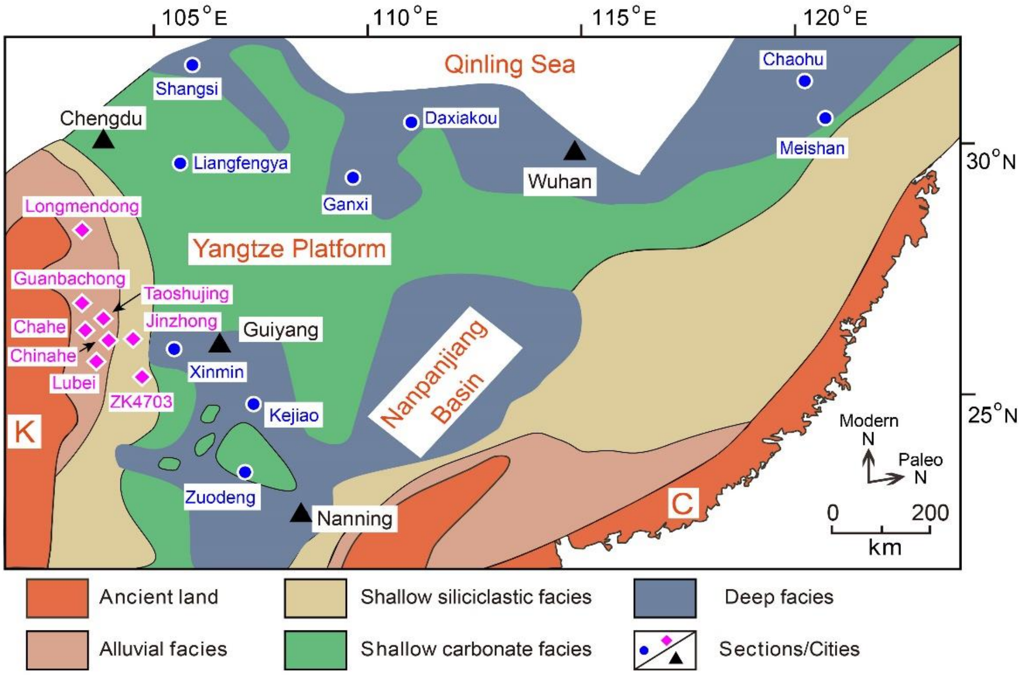


Supplementary Figure 1. Geographic distribution of the Permian–Triassic boundary sites in South China for which Hg studies have been undertaken[11]. Blue circles and red diamonds represent other marine and terrestrial sections, respectively. Abbreviations: C = Cathaysia Oldland, K = Kangdian Uplift. The sources for each site are given in Supplementary Table 1.

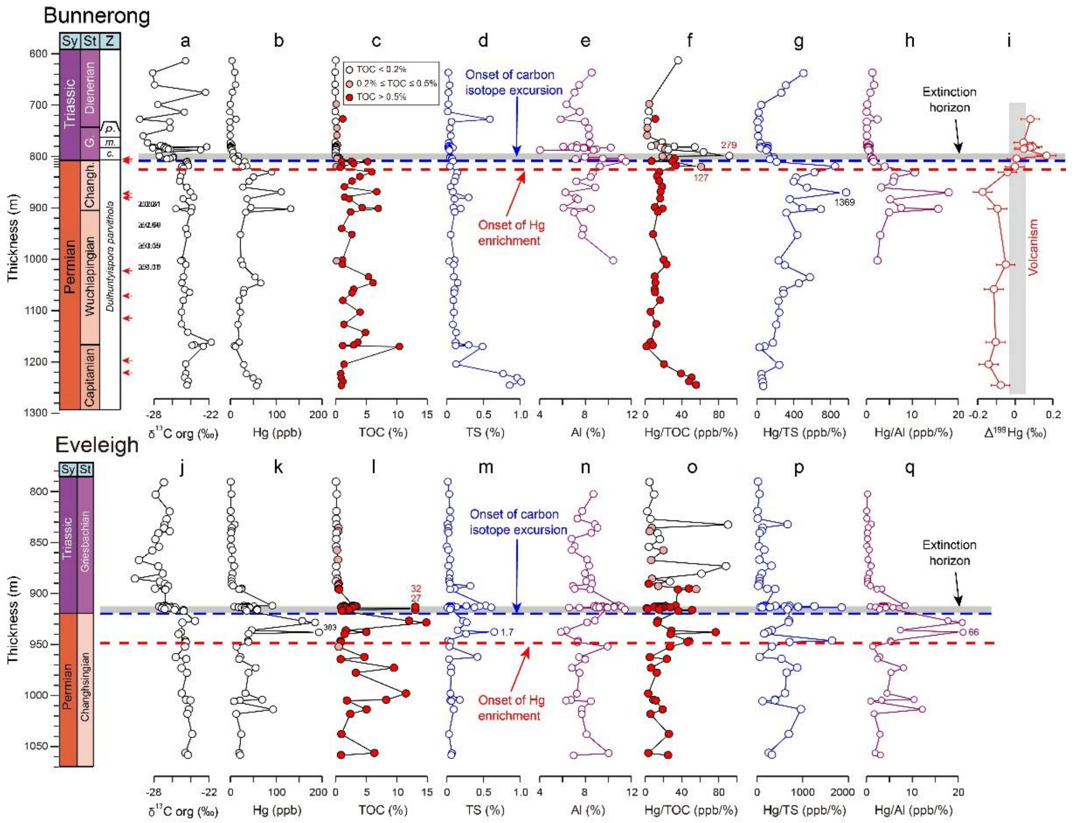


Supplementary Figure 2. Profiles of Bunnerong (above) and Eveleigh cores (below): (a, j) organic carbon isotope ($\delta^{13}C_{org}$, ‰); (b, k) mercury concentration (Hg, ppb); (c, l) total organic carbon concentration (TOC, %); (d, m) total sulfur concentration (TS, %); (e, n) aluminium concentration (Al, %); (f, o) ratio of mercury to total organic carbon (Hg/TOC, ppb/%); (g, p) ratio of mercury to total sulfur (Hg/TS, ppb/%); (h, q) ratio of mercury to aluminium (Hg/Al, ppb/ %); (i) mass independence fractionation of odd-Hg isotope ($\Delta^{199}Hg$, ‰). The red and blue dashed lines represent the onset of Hg enrichment and negative carbon isotope excursion, respectively. The horizontal gray rectangle represents the extinction interval for each section. The vertical gray rectangle in column i represents the $\Delta^{199}Hg$ of volcanic sources (+0.02 ± 0.06‰[12]). Open circles, pink-filled circles, and red-filled circles represent samples with TOC < 0.2%, 0.2–0.5%, and > 0.5% respectively in columns c, f, l, and o. Abbreviations: Sy = system, St = stage, Z = palynozone; Changh. = Changhsingian; G. = Griesbachian; *c.* = *Playfordiaspora crenulata*, m. = *Protohaploxypinus microcorpus*, *p.* = *Lunatisporites pellucidus*.

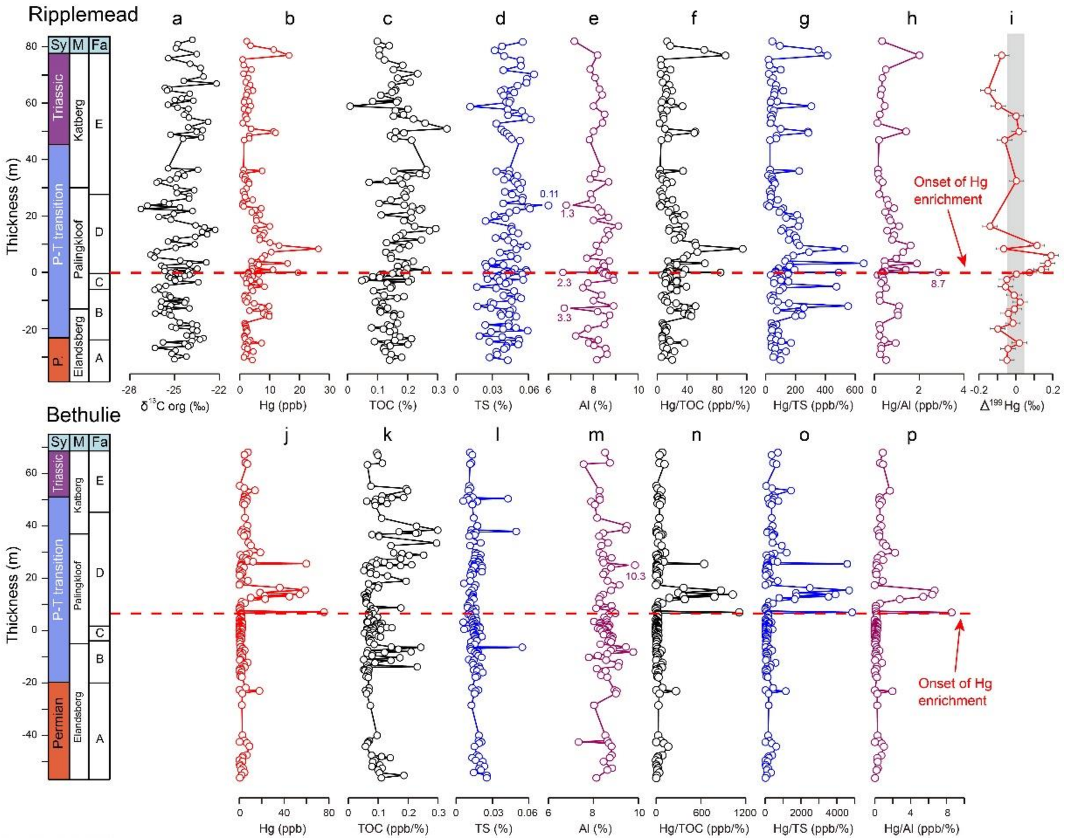


Supplementary Figure 3. Profiles of Ripplemead (above) and Bethulie sections (below): (a) organic carbon isotope ($\delta^{13}C_{org}$, ‰); (b, j) mercury concentration (Hg, ppb); (c, k) total organic carbon concentration (TOC, %); (d, l) total sulfur concentration (TS, %); (e, m) aluminium concentration (Al, %); (f, n) ratio of mercury to total organic carbon (Hg/TOC, ppb/%); (g, o) ratio of mercury to total sulfur (Hg/TS, ppb/%); (h, p) ratio of mercury to aluminium (Hg/Al, ppb/ %); (i) mass independence fractionation of odd-Hg isotope ($\Delta^{199}$Hg, ‰). The vertical gray rectangle in column i represents $\Delta^{199}$Hg values of volcanic source (+0.02 ± 0.06‰[12]). The red dashed line represents the onset of Hg enrichment. Abbreviations: Sy = system; M = member; Fa = facies.

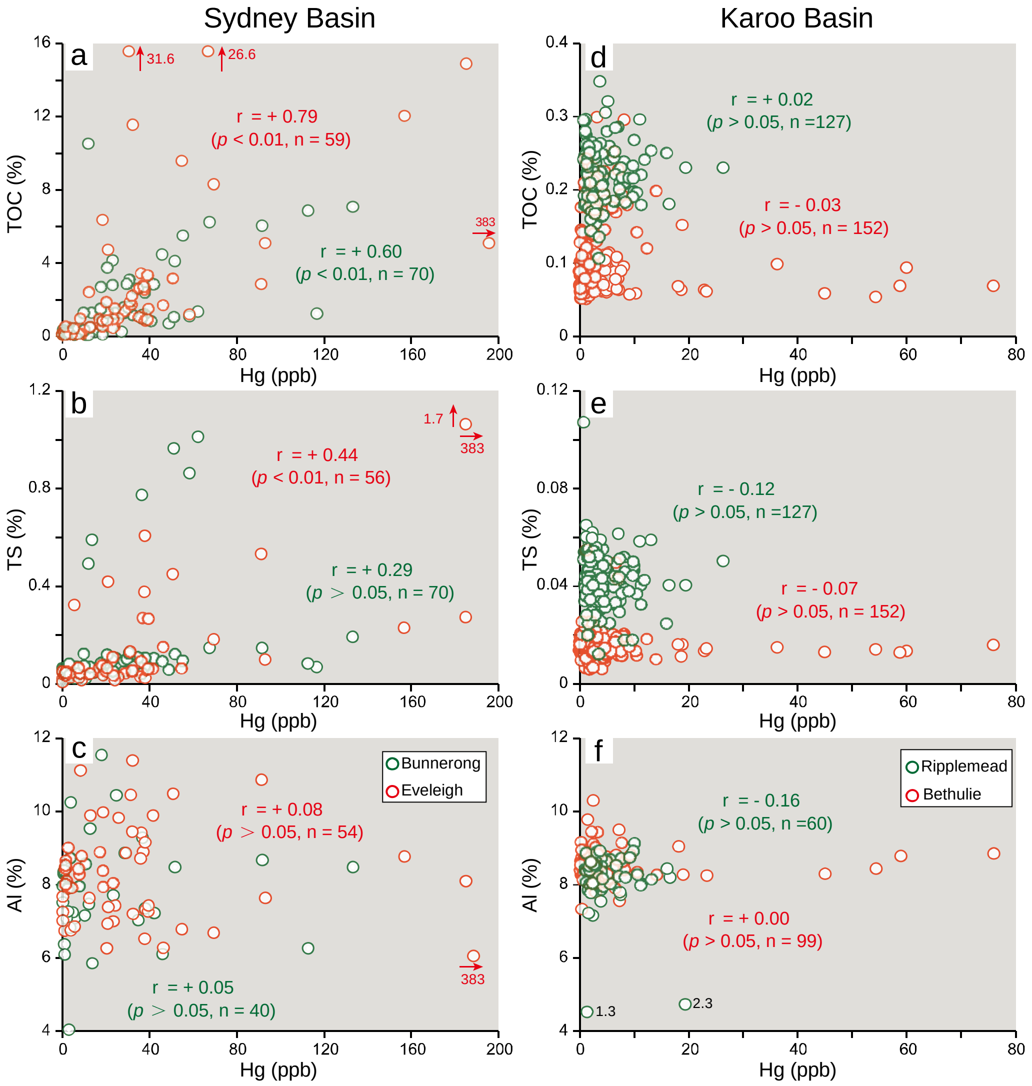


FIGURE S4

Supplementary Figure 4. Crossplots of TOC vs Hg, TS vs Hg, and Al vs Hg for the Bunnerong and Eveleigh cores in the Sydney Basin (a, b, c), and the Ripplemead and Bethulie sections in the Karoo Basin (d, e, f). *r* represents Pearson's correlation coefficient, *p* is the significance level and *n* represents the number of samples for each site.

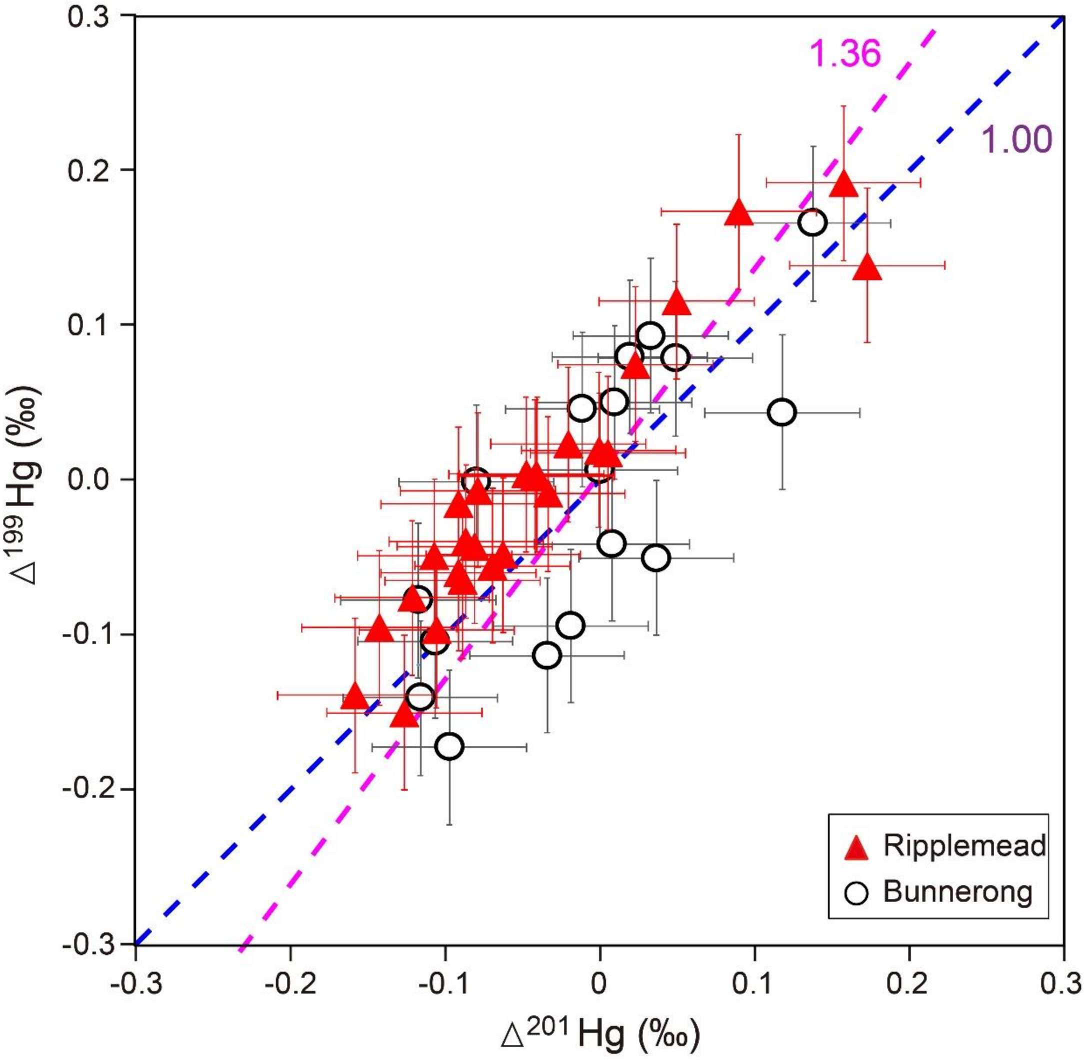


Supplementary Figure 5. Crossplot of $\Delta^{199}$Hg versus $\Delta^{201}$Hg for Ripplemead (red triangles) and Bunnerong (open circles). The dashed lines represent slopes of 1.00 (blue), and 1.36 (violet)[13]. The horizontal and vertical bars represent standard deviations (2σ) for $\Delta^{201}$Hg and $\Delta^{199}$Hg, respectively.

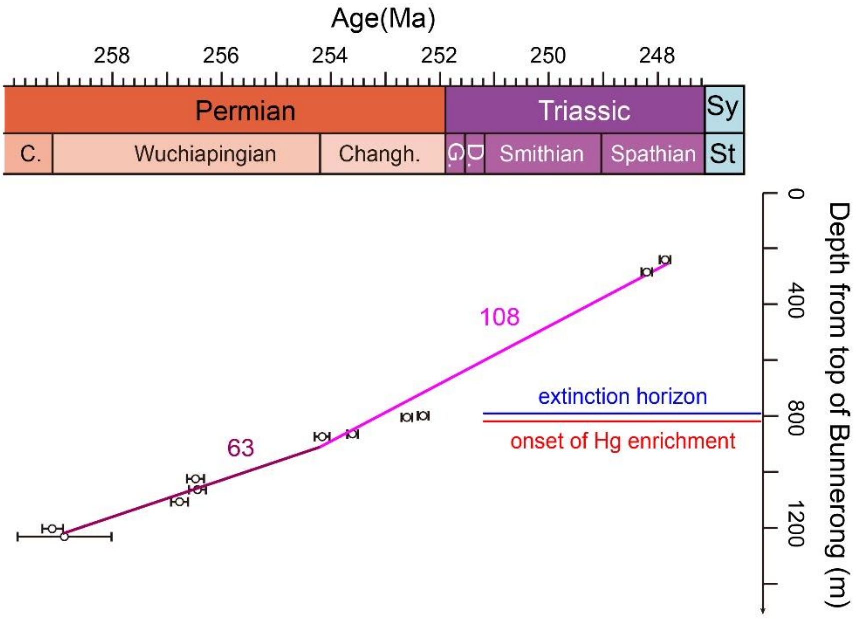


Supplementary Figure 6. Age-thickness model for the Bunnerong core in the Sydney Basin[9]. Linear sedimentation rates are given in units of m/Myr. The extinction interval and onset of Hg enrichments are located at ~800 m and ~820 m, respectively[8].

**Supplementary Table 1 Permian–Triassic boundary sections with published Hg data.**

| Section | Lithology | Environment | Location | Region | Sources |
|---|---|---|---|---|---|
| Buchanan Lake | shale | Marine | Canadian Arctic | Eastern Panthalassic Ocean | 14, 15 |
| Smith Creek | shale | Marine | Canada | Eastern Panthalassic Ocean | 16 |
| Festningen | shale | Marine | Spitsbergen | Eastern Panthalassic Ocean | 6 |
| Ursula Creek | shale | Marine | Canada | Eastern Panthalassic Ocean | 17, 18 |
| Opal Creek | shale | Marine | Western Canada | Eastern Panthalassic Ocean | 17 |
| Sheep Creek | mudstone, limestone | Marine | Northeastern Utah | Eastern Panthalassic Ocean | 19 |
| | | | | | |
| Dalongkou | mudstone | Continental | Northwest China | Northern Paleo-Tethys Ocean | 20 |
| | | | | | |
| Meishan | limestone | Marine | South China | Eastern Paleo-Tethys Ocean | 15, 17, 18, 21-23 |
| Chaohu | limestone | Marine | South China | Eastern Paleo-Tethys Ocean | 24, 25 |
| Daxiakou | shale, limestone | Marine | South China | Eastern Paleo-Tethys Ocean | 17, 21 |
| Ganxi | limestone | Marine | South China | Eastern Paleo-Tethys Ocean | 25 |
| Liangfengya | limestone | Marine | South China | Eastern Paleo-Tethys Ocean | 22 |
| Shangsi | mudstone, limestone | Marine | South China | Eastern Paleo-Tethys Ocean | 17, 25 |
| Xinmin | shale | Marine | South China | Eastern Paleo-Tethys Ocean | 17 |
| Kejiao | shale | Marine | South China | Eastern Paleo-Tethys Ocean | 17 |
| Zuodeng | limestone | Marine | South China | Eastern Paleo-Tethys Ocean | 26 |
| Longmendong | mudstone | Continental | South China | Eastern Paleo-Tethys Ocean | 27 |
| Guanbachong | mudstone | Continental | South China | Eastern Paleo-Tethys Ocean | 27 |
| Taoshujing | mudstone | Continental | South China | Eastern Paleo-Tethys Ocean | 27 |
| Chahe | siltstone | Continental | South China | Eastern Paleo-Tethys Ocean | 28 |
| Jinzhong | siltstone | Continental | South China | Eastern Paleo-Tethys Ocean | 28 |
| Chinanhe | mudstone | Continental | South China | Eastern Paleo-Tethys Ocean | 29 |
| Lubei | mudstone | Continental | South China | Eastern Paleo-Tethys Ocean | 20 |
| ZK4703 | mudstone | Continental | South China | Eastern Paleo-Tethys Ocean | 29 |
| | | | | | |
| Bálvány | limestone | Marine | Hungary | Western Paleo-Tethys Ocean | 17 |
| Bulla | limestone | Marine | northern Italy | Western Paleo-Tethys Ocean | 22 |
| Misci | marl | Marine | Southern Alps | Western Paleo-Tethys Ocean | 18 |
| Idrijca | carbonate | Marine | Slovenia | Western Paleo-Tethys Ocean | 18 |
| Rizvanuša | carbonate | Marine | Croatia | Western Paleo-Tethys Ocean | 18 |
| Zal | carbonate | Marine | Iran | Western Paleo-Tethys Ocean | 18 |

| | | | | | |
|---|---|---|---|---|---|
| Abadeh | limestone | Marine | Iran | Western Paleo-Tethys Ocean | 18 |
| | | | | | |
| Guryul Ravine | limestone | Marine | Northern India | Southern Paleo-Tethys Ocean | 23, 24 |
| Hovea-3 | limestone | Marine | Western Australia | Southern Paleo-Tethys Ocean | 18, 30 |
| Mud | limestone | Marine | India | Southern Paleo-Tethys Ocean | 26 |
| | | | | | |
| Gujo-Hachiman | chert, shale | Marine | Central Japan | Central Panthalassic Ocean | 17 |
| Ubara | chert, shale | Marine | Central Japan | Central Panthalassic Ocean | 17 |
| Akkamori | chert, shale | Marine | Northern Japan | Central Panthalassic Ocean | 17 |
| Waiheke | chert, shale | Marine | New Zealand | Central Panthalassic Ocean | 31 |

## Supplementary References


1. Grasby, S. E., Them II, T. R., Chen, Z., Yin, R., Ardakani, O. H. Mercury as a proxy for volcanic emissions in the geologic record. *Earth-Sci. Rev.* **196**, 102880 (2019).
2. Shen, J. et al. Sedimentary host phases of mercury (Hg) and implications for use of Hg as a volcanic proxy. *Earth Planet. Sci. Lett.* **543**, 116333 (2020).
3. Bower, J., Savage, K. S., Weinman, B., Barnett, M. O., Hamilton, W. P., Harper, W. F. Immobilization of mercury by pyrite ($FeS_2$). *Environ. Pollut.* **156**, 504-514 (2008).
4. Shen, J. et al. Mercury in marine Ordovician/Silurian boundary sections of South China is sulfide-hosted and non-volcanic in origin. *Earth Planet. Sci. Lett.* **511**, 130-140 (2019).
5. Farrah, H., Pickering, W. F. The sorption of mercury species by clay minerals. *Water Air Soil Pollut.* **9**, 23-31 (1978).
6. Grasby, S. E., Beauchamp, B., Bond, D. P., Wignall, P. B., Sanei, H. Mercury anomalies associated with three extinction events (Capitanian crisis, latest Permian extinction and the Smithian/Spathian extinction) in NW Pangea. *Geol. Mag.* **153**, 285-297 (2016).
7. Metcalfe, I., Crowley, J., Nicoll, R., Schmitz, M. High-precision U-Pb CA-TIMS calibration of Middle Permian to Lower Triassic sequences, mass extinction and extreme climate-change in eastern Australian Gondwana. *Gondwana Res.* **28**, 61-81 (2015).
8. Fielding, C. R. et al. Age and pattern of the southern high-latitude continental end–Permian extinction constrained by multiproxy analysis. *Nat. Commun.* **10**, 385 (2019).
9. Mays, C. et al. Refined Permian–Triassic floristic timeline reveals early collapse and delayed recovery of south polar terrestrial ecosystems. *Geol. Soc. Am. Bull.* **132**, 1489-1513 (2020).
10. Fielding, C. R. et al. Sedimentology of the continental end–Permian extinction event in the Sydney Basin, eastern Australia. *Sedimentology* **68**, 30-62 (2021).
11. Yin, H. F., Jiang, H. S., Xia, W. C., Feng, Q. L., Zhang, N., Shen, J. The end–Permian regression in South China and its implication on mass extinction. *Earth-Sci. Rev.* **137**, 19-33 (2014).
12. Yin, R. S. et al. Mercury isotopes as proxies to identify sources and environmental impacts of mercury in sphalerites. *Sci. Rep.* **6**, 18686 (2016).

13. Blum, J. D., Sherman, L. S., Johnson, M. W. Mercury isotopes in earth and environmental sciences. *Annu. Rev. Earth Planet. Sci.* **42**, 249-269 (2014).

14. Sanei, H., Grasby, S. E., Beauchamp, B. Latest Permian mercury anomalies. *Geology* **40**, 63-66 (2012).

15. Grasby, S. E. et al. Isotopic signatures of mercury contamination in latest Permian oceans. *Geology* **45**, 55-58 (2017).

16. Grasby, S. E., Sanei, H., Beauchamp, B., Chen, Z. Mercury deposition through the Permo–Triassic Biotic Crisis. *Chem. Geol.* **351**, 209-216 (2013).

17. Shen, J. et al. Evidence for a prolonged Permian–Triassic extinction interval from global marine mercury records. *Nat. Commun.* **10**, 1563 (2019).

18. Sial, A. et al. Globally enhanced Hg deposition and Hg isotopes in sections straddling the Permian–Triassic boundary: Link to volcanism. *Paleogeogr. Paleoclimatol. Paleoecol.* **540**, 109537 (2020).

19. Burger, B. J., Estrada, M. V., Gustin, M. S. What caused Earth's largest mass extinction event? New evidence from the Permian–Triassic boundary in northeastern Utah. *Glob. Planet. Change* **177**, 81-100 (2019).

20. Shen, J., et al. Mercury evidence of intense volcanic effects on land during the Permian–Triassic transition. *Geology* **47**, 1117-1121 (2019).

21. Wang, X. D. et al. Mercury anomalies across the end Permian mass extinction in South China from shallow and deep water depositional environments. *Earth Planet. Sci. Lett.* **496**, 159-167 (2018).

22. Kaiho, K., Aftabuzzaman, M., Jones, D. S., Tian, L. Pulsed volcanic combustion events coincident with the end–Permian terrestrial disturbance and the following global crisis. *Geology* **49**, 289-293 (2021).

23. Sial, A. N. et al. Hg isotopes and enhanced Hg concentration in the Meishan and Guryul Ravine Successions: proxies for volcanism across the Permian–Triassic Boundary. *Front. Earth Sci.* **9**, 651224 (2021).

24. Wang, X. D. et al. Global mercury cycle during the end–Permian mass extinction and subsequent Early Triassic recovery. *Earth Planet. Sci. Lett.* **513**, 144-155 (2019).

25. Shen, J. et al. Mercury fluxes record regional volcanism in the South China craton prior to the

end–Permian mass extinction. *Geology* **49**, 452-456 (2021).

26. Shen, J. et al. Mercury enrichments provide evidence of Early Triassic volcanism following the end–Permian mass extinction. *Earth-Sci. Rev.* **195**, 191-212 (2019)

27. Zhang, H. et al. Felsic volcanism as a factor driving the end–Permian mass extinction. *Sci. Adv.* **7**, eabh1390 (2021).

28. Wang, X. D. et al. Characteristics of Hg concentrations and isotopes in terrestrial and marine facies across the end–Permian mass extinction. *Glob. Planet. Change* **205**, 103592 (2021).

29. Chu, D. L. et al. Ecological disturbance in tropical peatlands prior to marine Permian–Triassic mass extinction. *Geology* **48**, 288-292 (2020).

30. Georgiev, S. V., et al. Late Permian–Early Triassic environmental changes recorded by multi-isotope (Re-Os-N-Hg) data and trace metal distribution from the Hovea-3 section, Western Australia. *Gondwana Res.* **88**, 353-372 (2020).

31. Grasby, S., Bond, D., Wignall, P., Yin, R., Strachan, L., Takahashi, S. Transient Permian–Triassic euxinia in the southern Panthalassa deep ocean. *Geology* **49**, 889-893 (2021).
