# Supplementary material for: Mercury evidence from southern Pangea terrestrial sections for end-Permian global volcanic effects
Source: Nat Commun. 2023 Jan 3;14:6. doi: 10.1038/s41467-022-35272-8 (PMC9810726; doi:10.1038/s41467-022-35272-8)
Supplement: Supplementary file 3 — Description of Additional Supplementary Files [file 41467_2022_35272_MOESM3_ESM.pdf]

Description of Additional Supplementary Files:

Supplementary Dataset 1: Geochemical dataset for the study sections.
